# Freeway Travel Speed Calculation Model Based on ETC Transaction Data

**DOI:** 10.1155/2014/174123

**Published:** 2014-12-15

**Authors:** Jiancheng Weng, Rongliang Yuan, Ru Wang, Chang Wang

**Affiliations:** Beijing Key Laboratory of Traffic Engineering, Beijing University of Technology, Beijing 100124, China

## Abstract

Real-time traffic flow operation condition of freeway gradually becomes the critical information for the freeway users and managers. In fact, electronic toll collection (ETC) transaction data effectively records operational information of vehicles on freeway, which provides a new method to estimate the travel speed of freeway. First, the paper analyzed the structure of ETC transaction data and presented the data preprocess procedure. Then, a dual-level travel speed calculation model was established under different levels of sample sizes. In order to ensure a sufficient sample size, ETC data of different enter-leave toll plazas pairs which contain more than one road segment were used to calculate the travel speed of every road segment. The reduction coefficient *α* and reliable weight *θ* for sample vehicle speed were introduced in the model. Finally, the model was verified by the special designed field experiments which were conducted on several freeways in Beijing at different time periods. The experiments results demonstrated that the average relative error was about 6.5% which means that the freeway travel speed could be estimated by the proposed model accurately. The proposed model is helpful to promote the level of the freeway operation monitoring and the freeway management, as well as to provide useful information for the freeway travelers.

## 1. Introduction

Automatic detection methods currently used in urban traffic flow collection usually include radar, laser, loop technologies based fixed detectors collection, or floating car (FC) method based on GPS technology. However, the fixed detectors are complex structures with high cost and inconvenience of installation and maintenance and only can capture the cross-section operation condition. Although the floating car data based collection system can get more accurate travel speed of road segments, there are still some problems such as insufficient number of floating vehicles and limited coverage as to the interregional and long-distance freeway. Zarrillo et al. pointed out that the automatic vehicle identification (AVI) technology is one possible solution to collect and measure traffic congestion at existing road facilities [1]. Xiao et al. thought that ETC (electronic toll collection) could significantly improve the handling efficiency of the toll station and the capacity traffic ability of toll road because of no parking troll collection [2]. As new traffic data, electronic toll collection (ETC) system recorded the time the vehicles enter and leave the freeway and the OD (origin-destination) information all day; this information is easy access, real-time, and high accuracy, and large-scale construction of the freeway ETC system is implemented around China recently. Additionally, the five provinces of Yangtze River Delta regions and five provinces including Beijing, Tianjin, Hebei, Shandong, and Shanxi provinces have been both realized cross-province ETC network charges [3]. With the expansion of ETC systems in all urban development scales, the number of users increases and the level of data makes the ETC transactions increase gradually. At some toll stations, ETC transactions made up nearly 30% of traffic flows and reached the minimum sample demand of traffic flow information collection. ETC data have become a new way of freeway traffic flow information collection.

Former studies on ETC transaction data mainly focus on freeway travel time forecasting and traffic flow statistics. Weng et al. explored the application feasibility of the ETC historical transaction data in freeway OD data, traffic flow, road of interval velocity, and so on and analyzed the related issues of transaction data and the data characteristics [4]. Myung et al. used *K* nearest neighbor model to establish a freeway travel time prediction model based on ETC transaction data and detector data [5]. Kwon and Varaiya presented a statistical model for electronic tags data and proved that there was an unbiased estimator of the OD matrix based on the method of moments. The algorithms put forward can be computed quickly and perform well under simulation compared with simpler estimators [6]. In the current study, attention has been focused on the access to speed, travel time, and traffic of the OD based on freeway ETC data, lacking research on more microscopic sections of freeway traffic information acquisition method.

In recent years, researchers put forward some techniques to estimate vehicle speed. Cathey and Dailey presented a novel method of automatically computing enough calibrated information for traffic surveillance cameras that were deployed on the freeway for congestion monitoring so that they could produce dependable speed estimates [7]. Doğan et al. also used video camera data to speed estimation; the accuracy of the method that they presented is approximately ±1.12 km/h. And they thought the sparse optical flow technique was a very effective technique for the speed estimation of the vehicles [8]. Ye et al. presented a new unscented Kalman filter method of speed estimation based on ingle-loop detectors data [9]. And, in 2009, Coifman and Kim fined a new method for estimating speed at single-loop detectors; the accuracy is closed to dual-loop detector's measurements [10]. Litzenberger et al. brought forward optical sensor system for traffic monitoring and vehicles speed estimation based on a neuromorphic “silicon-retina” image sensor, and a new algorithm was used for processing the asynchronous output data delivered by this sensor. The algorithm could measure velocities of vehicles in the range of 20 to 300 km/h on up to four lanes simultaneously [11]. However, despite the use in the collection of the vehicle speed, such advanced technology is rarely used for the study of the freeway travel information collection in China.

At present, the research on the real-time freeway traffic information collection model was mostly performed based on GPS technology and loop detectors data. Ferman et al. put forward that taking the vehicles with global positioning satellite (GPS) devices as probe cars will acquire more accurate and timely information, and have no large infrastructure construction and maintenance expenses. And they developed an analytical model that can examine the relationships between key system parameters [12]. Mihaylova et al. attempted to estimate the traffic state in freeway networks by particle filtering framework [13]. Jain and Coifman presented an analytical method to improve the accuracy of speed estimates from freeway traffic detectors by integrating information across lanes. This paper used a lane-by-lane basis using conventional aggregated flow and occupancy data obtained from single-loop detectors to improve speed estimates [14]. Bachmann et al. presented anonymous probe vehicle monitoring systems which are being developed to measure travel times on highways and arterials based on wireless signals available from technologies such as Bluetooth. And, through comparative experiment, they considered that fusing Bluetooth monitoring and loop detectors data can improve the accuracy of traffic estimates [15]. Despite the fact that the traffic information on the freeway can be more accurately obtained by the loop detector, Bluetooth, probe vehicle data, and so on are difficult to implement on the freeway which has the characteristics of long distance and interregionalism.

This paper is intended to analyze ETC data characteristics and to establish ETC data preprocessing procedure for freeway speeds extracting based on ETC transaction data. The paper used the speed of freeway road segment as calculation object and considered the applicability of the model under different levels of sample sizes and established segmented freeway travel speed calculation model to provide innovative solutions for freeway travel speed extraction.

## 2. Data Collection and Preprocess

### 2.1. ETC Transaction Data Foundations

Original ETC transaction data contains 74 fields, and it records the detail information of vehicles entering and leaving the freeway. The fields of data table mainly include the exit and entry plaza ID number, travel direction, exit and entry time, and vehicle type, which provide important database for computing the vehicle speed. It is shown in Table 1.

Besides ETC transaction data tables, the ETC transaction rate table was also used in the study, which contains the fields of exit and entry plaza ID number; charged mileage represents the location information of freeway toll stations. The distance between any two toll plazas can be calculated based on ETC transaction rate tables, as illustrated in Table 2.

### 2.2. Data Preprocessing

#### 2.2.1. Data Faults of Original ETC Transaction Data

Due to the system reasons, some errors and abnormal data can be found in the original ETC data, mainly including the following scenarios: (1) the presence of abnormal transaction time data: for example, time interval between exit and entry plaza is too short or too long. (2) “No card,” “negative balance,” and other special circumstances may occur when the vehicle goes through the toll station. (3) There are incomplete ETC transactions which were only partly performed by ETC Lane, including “ETC/MTC entry and MTC/ETC exit.” (4) There are some abnormal data with the speed excessively high or low due to the situation of vehicle stop or wrong system clock.

#### 2.2.2. Data Preprocessing

The preprocessing of ETC original data can provide a useful foundation for the model building. The object of data preprocessing is mainly the following: (1) high quality data can be got by deleting the error data and meaningless data; (2) it can obtain a single vehicle travel time of the sample vehicles corresponding to the exit and entry. For that purpose, the algorithm flow of ETC data preprocessing for extracting travel speed on the freeway includes key fields extraction, the erroneous data removing, valid data screening, data time division, and vehicle travel speed calculation.

#### 2.2.3. Data Filtering Rules

Meaningless or erroneous data in the ETC original transaction data will seriously influence the accuracy of the calculation model. The main objective of data screening is to discard unreasonable and erroneous data on the basis of following criteria:

1. the transaction data whose time interval between entrance and exit is more than 86,400 seconds (24 h) or less than 60 seconds should be erased;
2. if the exit time is earlier than the entry time, those transaction data should be deleted;
3. the transaction data with the same entrance and exit site should be removed;
4. the transaction data including open-charged freeway data should be deleted;
5. filtered out data abnormal data with “WORK-MODE (means normal records and special events)” field filled “0” (only the value of “0” stands for the normal data);
6. filtered out data with “DEALSTATUS” field filled “0X02” which means the vehicle's “entry by ETC and exit by ETC”;
7. filtered out data with “ENTRY_EXIT” field filled “1” which means it is exit data;
8. considering the speed threshold, the calculated vehicle travel speed lower than 5 km/h or higher than 120 km/h was discarded.

#### 2.2.4. The Time Interval of ETC Data Analyzing

**Figure 1:**
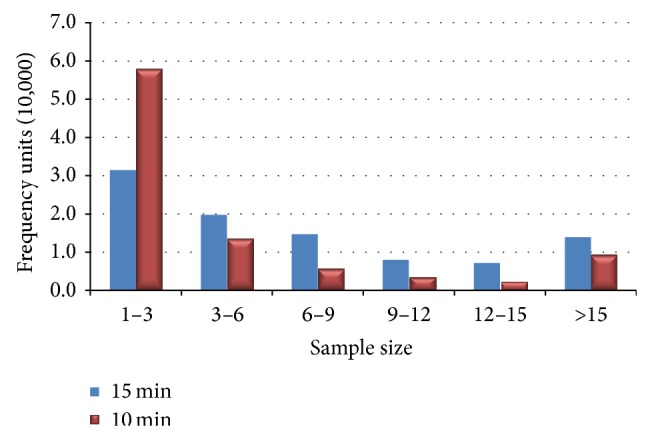
The sample sizes distribution in different time interval.

The time interval division of ETC transaction data should depend on the degree of variability of freeway traffic conditions, as well as sample size in a single analysis time period. According to the stability of the traffic flow, the study intended to select 10–15 minutes initially as the analysis period. In order to determine its rationality, the paper selected a whole working day randomly which contains all OD data in the whole network. 15 min and 10 min were considered to be the time interval, respectively, in this paper. And the paper counted the number of sample sizes for the same OD, as illustrated in Figure 1.

The figure generally demonstrated that under the 10 min interval, low sample size (less than 6) accounted for more than 78%. However, this ratio fell sharply to 54% when the time interval was 15 min. Consequently, the paper suggests 15 minutes as the time interval during the 6:00 AM tantamount to 11:00 PM and 1 hour as the remaining time analysis interval due to the small fluctuations of vehicle speed during the midnight hours.

#### 2.2.5. Travel Speed Calculating of Sample Vehicle

The average vehicle travel speed was calculated based on ETC data, and there were two steps as follows.

1. For the calculation of travel time of sample vehicle, the vehicle travel time can be calculated by the time difference of the entry time and exit time.
2. The travel speed of sample vehicles which travelled between certain freeways OD pair can be calculated by travel time and distance between the entry and exit toll stations which can be acquired from the ETC rate table.

## 3. Travel Speed Calculation Models

### 3.1. Road Segments Division

In order to reflect traffic state information on the freeway more accurately, freeway should as far as possible take the smallest unit. Toll station is the important node of the freeway network; road segment division can take the tolls station as the main node. This paper defined the section of same freeway directions between two adjacent tolls as road segment, which was the basic unit of travel speed calculation.

In particular, as some parts of the freeway have area with only one exit or entry toll station in special circumstances, there will be some gap if in accordance with the division of the freeway sections defined above. In view of this special situation, the virtual toll station was introduced in the research as supplement of the entrance or exit toll station.

### 3.2. Travel Speed Calculation Models for Road Segments

Considering that the ETC sample size of road segment may not be able to meet the minimum sample size requirement during a single time period, the paper established a dual-level travel speed calculation model according to sample size. These two situations were isolated by a sample size threshold in a single computation period (15 minutes). Travel speed of a road segment was the average value of all sample vehicles of the corresponding OD pair when the sample size was greater than the threshold conditions. However, when the sample was lower than the threshold conditions, the travel speed of sample vehicles of extensional OD pairs that contain calculation road segment was brought into the calculation. The model was built as follows:

(1) Vij=1n∑k=1nDjTik   n≥N0a1×vODij1×θ1+a2×vODij2×θ2⋯+az×vODijz×θz∑z=1zθz       n<N0,

where *V*_*ij*_ is the travel speed for *j* road segment at the *i* time interval; *D*_*j*_ is distance of *j* road segment; *T*_*ik*_ is travel time of the vehicle with the number *k* at the *i* time interval; *N*_0_ is the minimum sample size that is gained by data testing; *v*_OD_*ij*__^*z*^ is the average travel speed of the vehicle with the OD pair *z* containing *j* segment at the *i* time interval, *z* = 1,2,…, *z*, where  *z* is number of OD when sample size of road segment is deficient; *α*_*z*_ is the reduction coefficients for OD pair *z* speed, *z* = 1,2,…, *z*, where  *z* is number of OD when sample size of road segment is deficient; *θ*_*z*_ is the reliability weights for OD pair *z* speed, *z* = 1,2,…, *z*, where  *z* is number of OD when sample size of road segment is deficient.

Subsequently, the paper will specifically introduce the determination method for the minimum sample size, the reduction coefficient, and reliable weight for OD speed.

#### 3.2.1. Threshold of Sample Size

ETC sample size of the road segment in time interval affects the accuracy of monitoring travel speed and effectiveness seriously. When the ETC amount of data is sufficient, ETC vehicle travel speed can represent travel speed of road segment accurately. Compared with expressway, the freeway traffic flow was more continuous and the fluctuation of the velocity was much smaller. According to Xiong et al. that the city expressway in Beijing of five minutes per kilometer of floating vehicle minimum sample size was 7 to 9 and considered the research studied by Ferman and Tu et al. proposed that the floating car ratio should be no less than 3% in order to meet the accuracy of freeway speed monitoring [16, 17]; the paper recommended that the minimum ETC sample should be 12 in every analysis period of 15 minutes, which meant that the suggested value of *N*_0_ is 12.

#### 3.2.2. Reduction Coefficient for OD Speed

Because the travel speed of the vehicle may be affected by different traffic volumes and road conditions, OD speed that contains more than one road segment cannot be directly used for calculating the speed of certain road segment. In order to decrease the gap between the OD speed and the road speed, OD speed should be modified.

**Figure 2:**
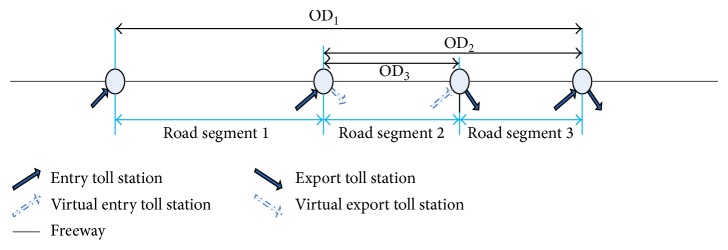
The diagram of relationship between road segments and OD pairs.

The study found that traffic flow operation states of the adjacent road segments had a strong correlation, and the relationship between them was rather steady (Figure 2). Therefore, reduction coefficient for OD speed was introduced in the models which can be obtained by analyzing relationship of historical traffic flow. First, the study calculated the travel speed of OD pair and the corresponding road segment based on the historical data. Then, speed reduction coefficient was the ratio of OD speed to road speed, and calculation formula should be

(2) $$\begin{array}{c} \alpha_{z}=\frac{V_{{\text{OD}}_{j}}^{Z}}{V_{j}}, \end{array}$$

where *α*_*z*_ is the reduction coefficient for OD pair *z*  speed to speed of road segment  *j*; *V*_*j*_ is historical travel speed for the road segment *j*; *v*_OD_*ij*__^*z*^ is the average travel speed of the vehicle with the OD pair *z* containing *j* segment, *z* = 1,2,…, *z*,  where  *z* is number of OD when sample size of road segment is deficient.

Research trained speed reduction coefficients in different period, and the results were shown in Table 3. From the training result, the speed reduction coefficient caused by OD to the road segment is not influenced by the time and the daily data is stable.

The relative error between OD speed and road segment speed was counted by the calculation method of speed reduction coefficient that was mentioned earlier, and the results were shown in Table 4. The results show that the OD speed can get closer to the speed of a road segment and discrete degree of the mean absolute error also declines through correcting by speed reduction coefficient.

#### 3.2.3. Reliability Weight for OD Speed

Despite the *α* being used to reduce the OD speed in the research, there still are certain errors between the OD speed and road segment speed because of speed fluctuation. The speed reliable weight indicator was introduced to evaluate the effect of OD on the reduction coefficient of velocity fluctuation. After the same value reduction, OD with velocity fluctuations will bring greater error, thus giving low credibility. So the second parameter *θ* was introduced into the model.

Reliability weight can be given by *θ* to the OD speed with smaller error which is stable after the reduction. In this way, the calculation error caused by the fluctuation of speed can be reduced. Therefore, the mean absolute error between speed of road segment and OD speed that has been reduced based on historical data and data experiments was calculated, thus giving different reliability weight for OD speed according to the mean absolute error. The paper supposed that the given speed reliable weight of road section itself was 1.0, and the total weights of related multiple OD pairs speeds of calculated road section were 1.0. The specific calculation formula was as follows:

(3) $$\begin{array}{c} \theta_{z}=\frac{1/m_{z}}{\sum_{i=1}^{i}1/m_{i}}\times 1, \end{array}$$

where *θ*_*z*_ is the reliability weights for OD pair *z* speed, *z* = 1,2,…, *z*, where *z* is number of OD when sample size of road segment is deficient; *m* is mean absolute error between historical OD speed and road segment speed.

## 4. Verification of Speed Calculation Model

The accuracy of the proposed travel speed calculation model was tested by comparing the calculated speed with the observed speed on freeway. The moderate drivers were selected to conduct the field experiments and they drive through the experimental road segment at an average speed of traffic flow. The study selected 12 road segments, respectively, belonging to Beijing-Harbin freeway, Beijing-Kaifeng freeway, and Beijing-Tibetan freeway which have different levels of daily traffic volume. The verification experiments were conducted both during peak hours (17:30–19:00) and off-peak (14:00–15:30) hours.

The error and prediction precision of the calculation models are evaluated by three indicators including the mean absolute error (MAE), mean relative error (MRE), and root mean square error (RMSE). The former two indexes are used to describe the basic error of calculating speed and the actual speed. The third indicator is utilized to characterize the discrete degree of calculated speed and the actual data.

The calculation formulas of error evaluation indicators were shown as follows:

(4) $$\begin{array}{c} \text{MAE}=\frac{1}{n}\overset{n}{\underset{i=1}{\sum }}\left(\left|V_{\text{calculation}\;i}-V_{\text{true}\;i}\right|\right),\\ \text{MRE}=\frac{\left(1/n\right)\sum_{i=1}^{n}\left|V_{\text{calculation}\;i}-V_{\text{true}\;i}\right|}{V_{\text{true}\;i}},\\ \text{RMSE}=\sqrt{\frac{1}{n}\overset{n}{\underset{i=1}{\sum }}{\left[\left|V_{\text{calculation}\;i}-V_{\text{true}\;i}\right|-\text{AAE}\right]}^{2}}. \end{array}$$

According to the calculation formula of each indicator, results are shown in Table 5.

The result shows that the overall error of the model that the study presented is small, and the model has a higher precision. In the peak period and off-peak period, the average absolute error is less than 5 km/h, respectively, 4.11 km/h and 4.93 km/h, and the average relative error is about 6.5%. Two indexes are the acceptable level of error. Median square deviations are, respectively, 2.38 km/h and 3.39 km/h, and the precision of the results indicates that it remains stable. The travel speed model proposed by the study is of better applicability; it can satisfy the requirements of the peak and off-peak travel speed collection, especially that the root mean square error is smaller in the peak hours; it indicates that the fluctuation of the speed calculation error during the peak hours is smaller.

## 5. Conclusions and Further Works

Based on the analysis of the ETC transaction data mining application and the freeway information extraction, some important conclusions can be drawn as follows.

1. ETC transaction data provides a feasible method for freeway travel speed extraction. Considering the condition of the ETC transaction ratio, 15 minutes is usually a proper time interval to discover disclose and analyze the dynamic traffic state of the freeway.
2. Considering that ETC sample size of the road segment in a single time period was different, the study built a dual-level travel speed calculation model under different samples levels. The study used the ETC transaction data of OD pairs that contain calculation road segment to calculate travel speed of a road segment. And the speed reduction coefficient and speed reliability weight were created; they can make the OD speed more accurate in the calculation of travel speed of a road segment.
3. Based on collected freeway road segment travel speed, the calculation models proposed in this paper were validated. The results show that the average absolute error of the peak and off-peak is about 4.5 km/h, mean relative error the average relative error is about 6.5%, and the mean square deviations are 2.38 km/h and 3.39 km/h. It shows that travel speed calculation model is feasible and suitable for the peak period and off-peak period, and the errors have no significant effect on the travel speed calculation.

The ETC transaction data can only record the time entry and depart freeway of vehicles. In the future study, the ETC transaction data and the other data sources can be utilized together to extract the freeway traffic flow operation information, such as the fixed detector data, floating vehicle data, and video data. The multisource data fusion based algorithm will further enhance the accuracy and reliability of freeway operation state collection.

## Figures and Tables

**Table 1:** The descriptive of the ETC transaction data.

Fields	PLAZAID	Direction	ENTRY_EXIT	CAR_SERIAL	CREATED	EN_PLAZAID	EN_TIME
Explanatory note	Exit plaza ID	1: up; 0: down	0: entry; 1: exit	License plate	Exit time	Entry plaza ID	Entry time
Sample data	100124	1	0	PA1234	2013/9/1 0:08:01	100122	2013/9/1 0:01:23
	100433	0	0	FZ1234	2013/9/1 0:17:18	100711	2013/9/1 0:11:01

**Table 2:** The distances between toll stations.

S.N.	Exit plaza ID	Entrance plaza ID	Path ID	Distance (m)	Exit plaza name	Entrance plaza name
1	100016	100017	43800001	4320	Liuyuanqiao exit	Shunyi entry
2	100012	100017	43800002	8610	Zhangxizhuang exit	Shunyi entry

**Table 3:** The result for training speed reduction coefficient *α*.

Speed reduction coefficient	Peak periods			Off-peak periods			All day		
Test data group	Average value of *α*	STDEV	Sample size	Average value of *α*	STDEV	Sample size	Average value of *α*	STDEV	Sample size
1	0.83	0.02	6	0.84	0.02	8	0.83	0.02	14
2	0.95	0.04	6	0.94	0.03	8	0.94	0.03	14
3	0.95	0.07	6	0.94	0.02	8	0.94	0.05	14
4	0.86	0.03	6	0.87	0.02	8	0.87	0.02	14

**Table 4:** Velocity error comparison of before and after reduction.

Road segment	Without reduction		With reduction		Sample size
	Mean absolute error (km/h)	Error variance (km/h)	Mean absolute error (km/h)	Error variance (km/h)	
1	8.92	6.15	3.57	3.45	84
2	14.98	8.69	5.36	3.51	84

**Table 5:** The error analysis results of speed calculation models.

Error analysis	Mean absolute error (km/h)	Mean relative error (%)	The mean square error of error (km/h)
Peak periods	4.11	6.42	2.38
Off-peak periods	4.93	6.74	3.39
